# Machine learning methods to discover hidden patterns in well‐being and resilience for healthy aging

**DOI:** 10.1111/jnu.13025

**Published:** 2024-09-09

**Authors:** Robin R. Austin, Ratchada Jantraporn, Martin Michalowski, Jenna Marquard

**Affiliations:** ^1^ School of Nursing, University of Minnesota Minneapolis Minnesota USA; ^2^ Institute for Health Informatics Minneapolis Minnesota USA

**Keywords:** consumer‐generated health data, machine learning, resilience, whole person health

## Abstract

**Background:**

A whole person approach to healthy aging can provide insight into social factors that may be critical. Digital technologies, such as mobile health (mHealth) applications, hold promise to provide novel insights for healthy aging and the ability to collect data between clinical care visits. Machine learning/artificial intelligence methods have the potential to uncover insights into healthy aging. Nurses and nurse informaticians have a unique lens to shape the future use of this technology.

**Methods:**

The purpose of this research was to apply machine learning methods to MyStrengths+MyHealth de‐identified data (*N* = 988) for adults 45 years of age and older. An exploratory data analysis process guided this work.

**Results:**

Overall (*n* = 988), the average Strength was 66.1% (SD = 5.1), average Challenges 66.5% (SD = 7.5), and average Needs 60.06% (SD = 3.1). There was a significant difference between Strengths and Needs (*p* < 0.001), between Challenges and Needs (*p* < 0.001), and no significant differences between average Strengths and Challenges. Four concept groups were identified from the data (Thinking, Moving, Emotions, and Sleeping). The *Thinking* group had the most statistically significant challenges (11) associated with having at least one *Thinking* Challenge and the highest average Strengths (66.5%) and Needs (83.6%) compared to the other groups.

**Conclusion:**

This retrospective analysis applied machine learning methods to de‐identified whole person health resilience data from the MSMH application. Adults 45 and older had many Strengths despite numerous Challenges and Needs. The Thinking group had the highest Strengths, Challenges, and Needs, which aligns with the literature and highlights the co‐occurring health challenges experienced by this group. Machine learning methods applied to consumer health data identify unique insights applicable to specific conditions (e.g., cognitive) and healthy aging. The next steps involve testing personalized interventions with nurses leading artificial intelligence integration into clinical care.

## INTRODUCTION

Healthy aging, defined as the preservation of functional ability and well‐being with older age, is essential for maintaining the overall quality of life for a growing global older adult population (Rudnicka et al., 2020). Current recommendations to support healthy aging include the use of an integrative whole person approach that includes a person's strengths in addition to their challenges (Langevin et al., 2024; Wu & Sheng, 2019). Whole person health takes into account an individual's environment, physical health, psychosocial aspects, and health behaviors. Whole person health also includes strengths (or resilience) (Langevin et al., 2024). Strengths are defined as assets of individuals to maintain or improve their well‐being in the face of short and long‐term stressors (Aungst et al., 2019). Emerging research has shown an individual's resilience may be a strong indicator of healthy aging. For example, individuals with higher resilience can achieve positive health outcomes despite health challenges (Lysne, 2020; Yeung et al., 2012). This research study moves toward a comprehensive whole person approach to address the interrelating factors that can influence healthy aging (Ziglio et al., 2017; Langevin et al., 2024; National Academies of Sciences, Engineering, and Medicine, et al., 2023; National Center for Complementary and Integrative Health, 2021).

Consumer‐generated whole person health data can provide comprehensive insights into individuals' overall well‐being, enabling personalized treatment plans and interventions that address both physical and social drivers of health. Furthermore, MyStrengths+MyHealth (MSMH), a (mHealth) application, developed in 2017, as a means to capture comprehensive holistic health assessment that also includes a strengths perspective (Bakken, 2021a; Hsueh et al., 2017). Since its development, MSMH has been used in various community settings and research projects to capture consumer‐generated whole person health and strengths data from community participants (Agboola et al., 2022; Austin et al., 2023; Austin, Jones, et al., 2022; Austin, Mathiason, et al., 2021; Rajamani et al., 2022). Data from MSMH hold potential to provide insights into healthy aging such as sleep issues, income challenges, or unmet needs related to mental health.

**FIGURE 1:**
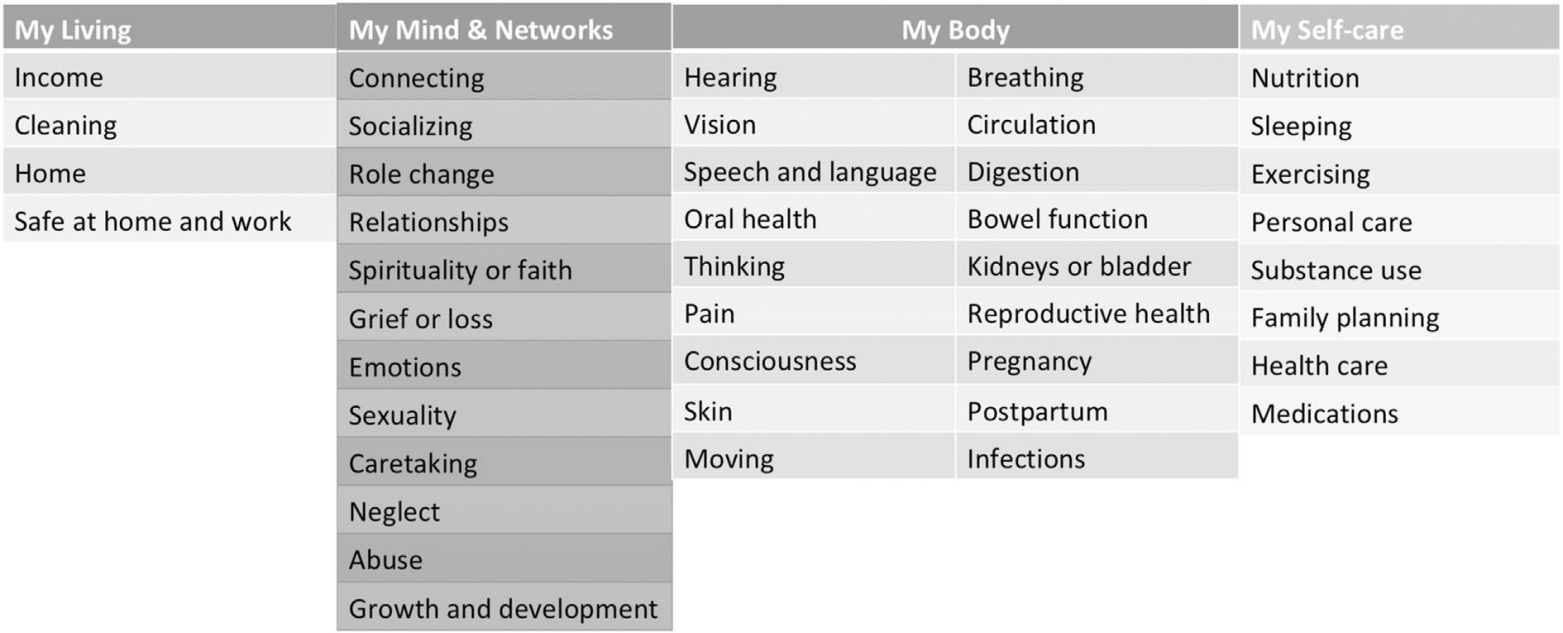
MyStrengths+MyHealth concepts.

MSMH leverages the rigor of the Omaha System, a multidisciplinary standardized health terminology that encompasses whole person health across four domains with 42 discrete concepts (Austin, Mathiason, et al., 2021). The Omaha System enables a framework for whole person health as it describes and quantifies health in 42 discrete, taxonomic concepts arranged within four domains: Environmental (four concepts), Psychosocial (12 concepts), Physiological (18 concepts), and Health‐related behaviors (eight concepts) (Martin, 2005; Monsen et al., 2014, 2021). The Omaha System framework enables the use of standardized terminology and supports data standards within a consumer application (Austin, Mathiason, et al., 2021). In the development of MSMH, researchers translated all the terms of the Omaha System to the Simplified Omaha System Terms (SOST) using expert‐ and community‐validated methods as described in previous research (Austin, Martin, et al., 2022). The SOST terms have been validated at the fifth‐grade reading level (Austin, Martin, et al., 2022). In MSMH, the Omaha System domains were renamed My Living, My Mind and Network, My Body, and My Self‐care (Figure 1).

**FIGURE 2:**
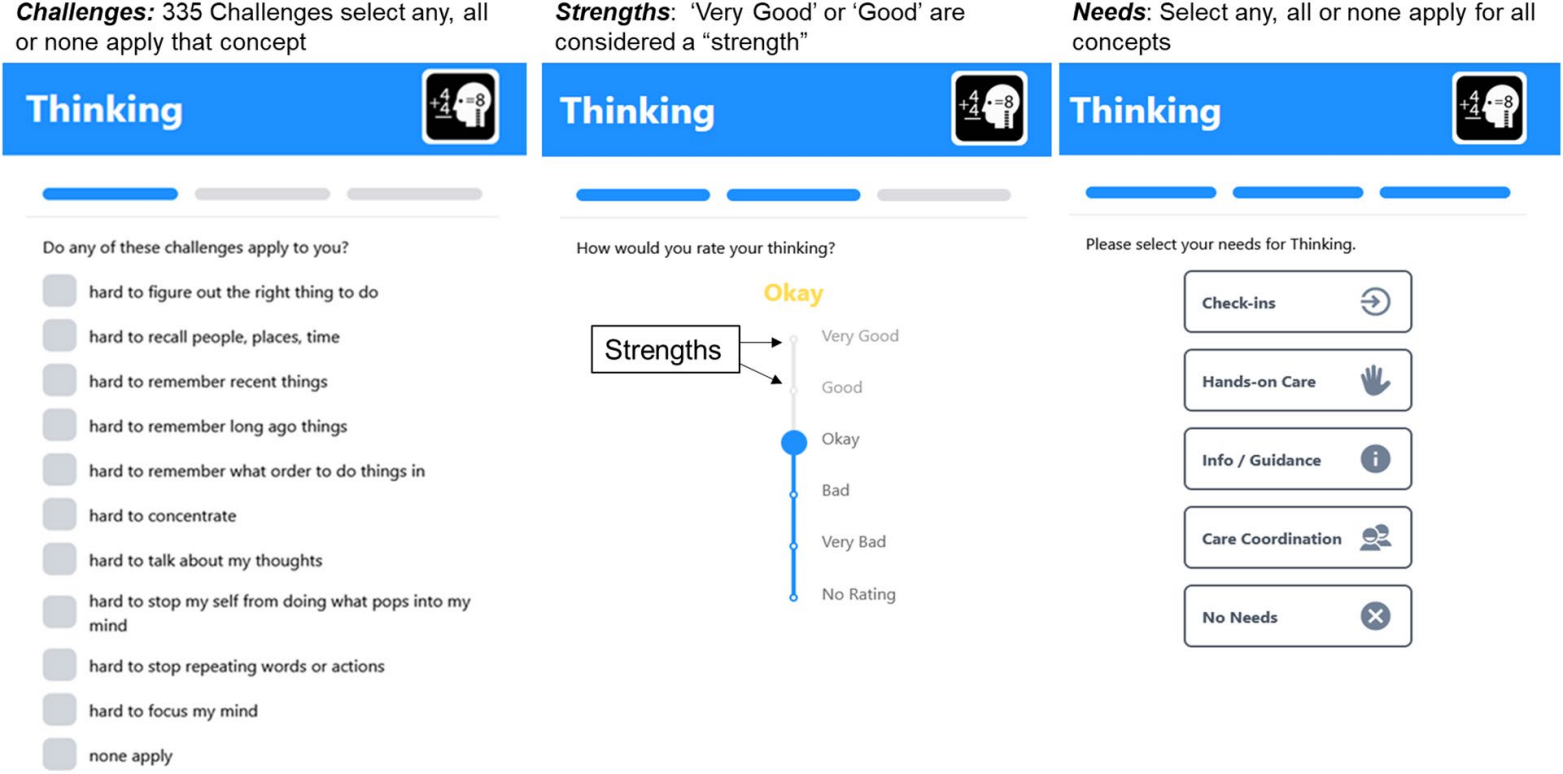
MyStrengths+MyHealth screenshot of the thinking concept.

In MSMH, Strengths are reported using the Omaha System Problem Rating Scale for Outcomes Status scale responses (“Strengths” = a score of 4–5 of 5). Strengths are identified if participants self‐report a status score of “4” (a “Good” rating) or “5” (a “Very good” rating) on a scale of 1 (“Very bad”)–5 (“Very good”). The Omaha System Problem Classification Scheme concept signs/symptoms “Challenges” in MSMH are the unique signs/symptoms associated with a specific concept and range from 2 to 16 Challenges per concept. In MSMH, users can select which Challenges may apply or if none apply select “None apply.” The Omaha System Intervention Scheme, “Needs” in the MSMH, describes problem‐specific actions and activities. Users can select any, all, or none from the four categories: Teaching, guidance, and counseling (MSMH=Info/guidance); Treatments and procedures (MSMH=Hands‐on care); Case management (MSMH=Care coordination); Surveillance (MSMH=Check‐ins); or No Needs if none applicable (Austin, Mathiason, et al., 2021) (Figure 2).

The use of data from a tool such as MSMH facilitates the exploration of consumer‐generated whole person health data and resilience through data‐driven artificial intelligence (AI) approaches. AI methods can help examine these complex whole person health factors that can influence aging, and emerging research suggests AI methods have the potential to fill a gap in unmet care needs of older adults (Ma et al., 2023). The purpose of this research is to apply machine learning methods, one domain of AI, to whole person health data from a large sample of older adults, to explore social and environmental factors associated with healthy aging. The use of machine learning methods can generate new insights into whole person health (e.g., physical and social drivers of health) and resilience (Ma et al., 2023). These new insights can then be used by nurses to address potentially unmet needs of older adults. The combination of machine learning methods applied to consumer‐generated health data is a unique combination that can generate new evidence, provide context from the consumers' perspective, and support personalized approaches for healthy aging (Bakken, 2021b, p. 20). Nurses and nurse informaticians play a vital role in applying machine learning methods and leveraging technology to enhance interventions for healthy aging (Ronquillo et al., 2021; von Gerich et al., 2022).

### Design

The purpose of this research was to apply machine learning methods to de‐identified whole person health data from the MSMH application to explore potential hidden patterns of self‐reported Strengths, Challenges, and Needs for adults ages 45 years and older. Our specific aims were to: (1) Apply machine learning methods to MSMH data using an exploratory data‐driven approach to generate insights into what aspects of whole person health are related to healthy aging; and (2) Examine differences in self‐reported whole person health data (Strengths and Needs) for adults 45 years and older.

## METHODS AND METHODS

### Data description

**FIGURE 3:**
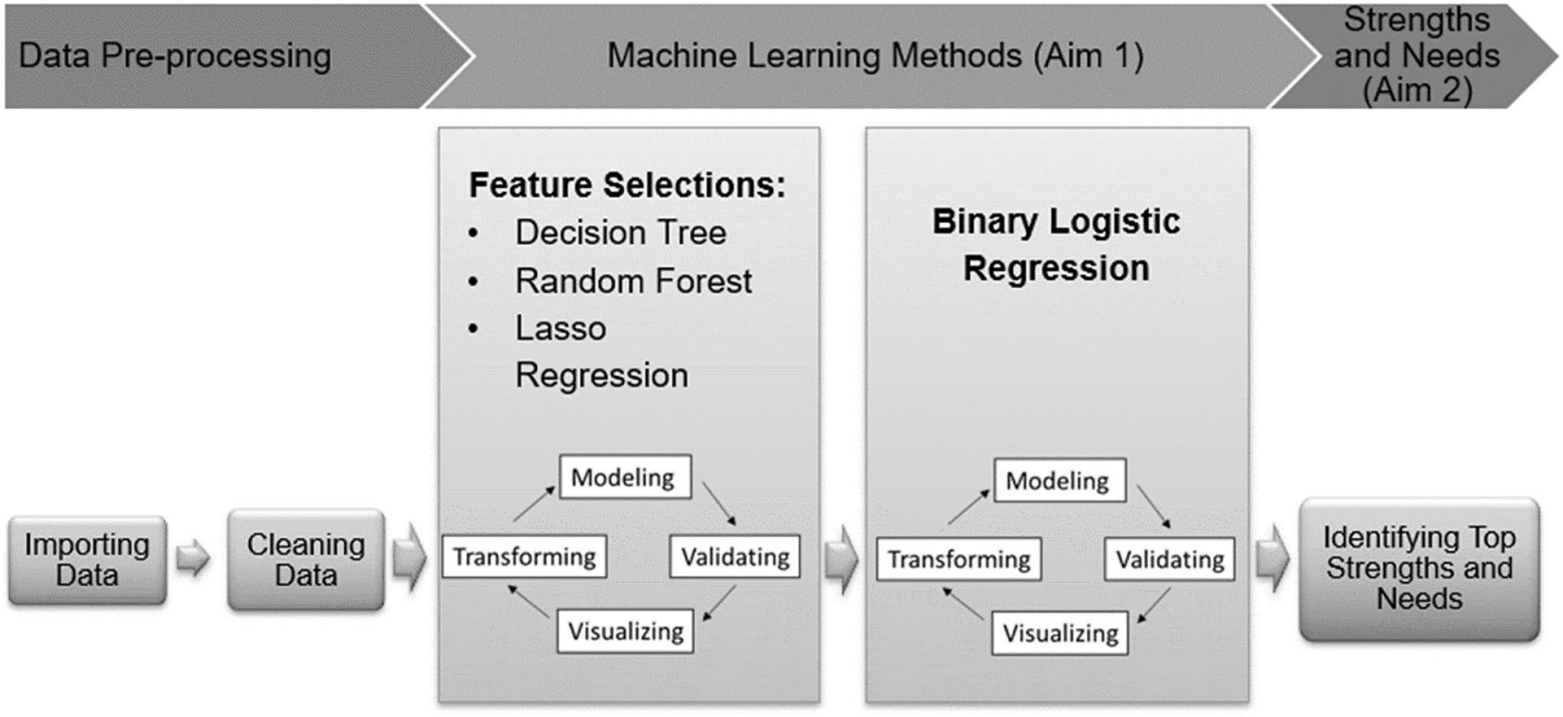
Data analytical process with associated study aims.

This study used de‐identified data from the MyStrengths+MyHealth (MSMH) app and was approved by the University of Minnesota Institutional Review Board (IRB). All de‐identified data were compliant with the Health Insurance Portability and Accountability Act (HIPAA). MSMH data were collected in various community settings between August 2019 and September 2023 and collected via phones, iPads, or tablets across various community settings. Participants were community‐dwelling adults voluntarily completing the MSMH application. The raw dataset included 1224 respondents and 549 variables for each respondent [demographics (5), Strengths (42), Challenges (334), and Needs (168)]. Demographic data included: Age (grouped as 18–24, 25–44, 45–64, and 65+ years); Gender (self‐identified as Male, Female, Non‐binary, Transgender); Marital status (Divorced, Married, Other, Partnered, Single, Widowed); Race (Asian, Black/African American, Native American/Alaskan, Native Hawaiian/Pacific Islander, Two or more, Other, White); and Ethnicity (Hispanic/Latinx, Non‐Hispanic/Non‐Latinx). All data were analyzed using SPSS and R version 4.3.2. We followed a systematic approach for our analysis (Figure 3).

### Data cleaning, transforming, and pre‐processing

#### Data cleaning

Data cleaning included handling missing values and eliminating irrelevant data (e.g., participants younger than 45 years old), 9% of the total data contained missing values, and those observations with missing values were deleted. We excluded seven concepts based on low response rates (sparse data) or irrelevance to the population: *Spirituality or Faith*, *Growth and Development*, *Consciousness*, *Reproductive Health*, *Pregnancy*, *Postpartum*, and *Family Planning*. The final dataset include 988 respondents and 488 variables [Demographics (5), Strengths (36), Challenges (303), and Needs (144)].

#### Transforming

Transforming the data included converting Strengths data from ordinal to nominal data. Strengths data were reported on a 5‐point Likert scale, ranging from 1 (very bad) to 5 (very good) and any concept that received a rating of 4 (good) or 5 (very good) was considered a strength. To convert the data into binary form, we labeled concepts that received a rating of 4 or 5 as “Yes” (1), indicating that the respondents have strengths in that concept. The concepts with a rating less than 4 were labeled as “No” (0), indicating no strength in that concept. Challenges and Needs were already in nominal form, where “Yes” (1) indicated the presence of a challenge or need, and “No” (0) indicated the absence of a challenge or need.

#### Pre‐processing

Using the final dataset (*N* = 988), we pre‐processed the data using an iterative exploratory data analysis approach and applied Principal Component Analysis (PCA), Multiple Correspondence Analysis (MCA), and Decision Tree Analysis to the entire data and subsets of the data (e.g., Challenges only or Strengths only) (Greenacre & Blasius, 2006; Rigner, 2008). To further reduce the dimensionality of the data, we focused on the Challenges only data subset, and selected four MSMH challenges. The challenges were determined based on previous MSMH research, frequency in the current MSMH data, and common health issues identified in adult populations (Austin, 2018; Austin, Monsen, & Alexander, 2021; Rajamani et al., 2022). The four Challenges included: *Thinking* (Omaha System term: Cognition), *Moving* (Omaha System term: Neuro‐musculo‐skeletal function), *Emotions* (Omaha System term: Mental health), and *Sleeping* (Omaha System term: Sleep and rest patterns) (Table 1).

**TABLE 1:** Thinking, moving, emotions, and sleeping concepts and associated challenges.

Thinking (10)	Moving (13)	Emotions (16)	Sleeping (8)
Hard to figure out the right thing to do	Hard to extend or move arms or legs fully	Very sad, hopeless	Hard to keep from bothering others
Hard to recall people, places, time	Weak muscles	Fearful	Wake up a lot at night
Hard to remember recent things	Not coordinated	Not interested in taking care of myself	Sleep walk
Hard to remember long ago things	Low muscle tone	Hard to concentrate	Cannot get to sleep
Hard to remember what order to do things in	Tight muscles	Nothing excites me	Nightmares
Hard to concentrate	Numbness	Strongly annoyed and acting out	Not enough sleep
Hard to talk about my thoughts	Tingling	Hard to not repeat things I do	Stop breathing during sleep
Hard to stop myself from doing what pops into my mind	Hard to keep my balance	Hard to manage my stress	Snore
Hard to stop repeating words or actions	Hard to walk	Angry	
Hard to focus my mind	Hard to go from bed to chair	Tired	
	Broken bones	Hard to understand real life	
	Tremors or seizures	See or hear things that others cannot	
	Feel too hot or too cold	I think about killing myself or others	
		Self‐harm	
		Mood swings	
		Flash‐backs	

### Machine learning methods (Aim 1)

For feature selection, we used machine learning methods to reduce the dimensionality of our dataset and as a primary strategy to mitigate overfitting during exploratory data analysis. By systematically identifying and selecting the most informative features, we aimed to reduce data complexity and focus on the most relevant variables. This approach enhanced interpretability and reduced the risk of overfitting by excluding potentially noisy or irrelevant variables. In the initial step, we explored a range of methods, including Decision Tree, Random Forest, and Lasso Regression, specifically using the *Thinking* concept within the cleaned dataset containing solely Challenges. This step was designed to identify the most influential independent variables (Challenge variables) that are associated with the *Thinking* concept. To ensure accuracy, we divided the dataset into training (80%) and testing (20%) datasets. We used the training dataset to assess method accuracy through a validation process. We conducted validation using the F‐1 (harmonic mean) score. This metric considers both precision and recall and provides a thorough evaluation of the effectiveness of the method. The highest F‐1 score was identified as the best‐performing one and selected for further detailed analysis. Among selected feature selection methods, Random Forest was the top‐performing.

To examine the data using binary logistic regression, we use the precise subset of Challenges variables derived from the random forest analysis. This enabled us to examine the connections between the dependent variable, “*Thinking*,” and the other variables in the MSMH dataset. We used Random Forest to iteratively assess the impact of adding or excluding independent variables and refine the subset of relevant variables that would yield the highest F‐1 score when integrated with binary logistic regression. In this process, we also divided the dataset into training (80%) and testing (20%) datasets with repeated training and testing while fitting the binary logistic regression. This thorough exploration and refinement of a relevant subset of variables ensured that binary logistic regression fit with the most significant predictors. We repeatedly performed feature selection using random forest before fitting the binary logistic regression to address the challenges related to *Moving*, *Emotions*, and *Sleeping* concepts.

### Examine differences strengths and needs (Aim 2)

Once the key challenges were identified (*Thinking*, *Moving*, *Emotions*, and *Sleeping*), we examined prevalent Strengths and Needs for each group to gain a deeper insight into coping mechanisms and potential needs to support healthy aging.

## RESULTS

### Data description and machine learning methods (Aim 1)

**FIGURE 4:**
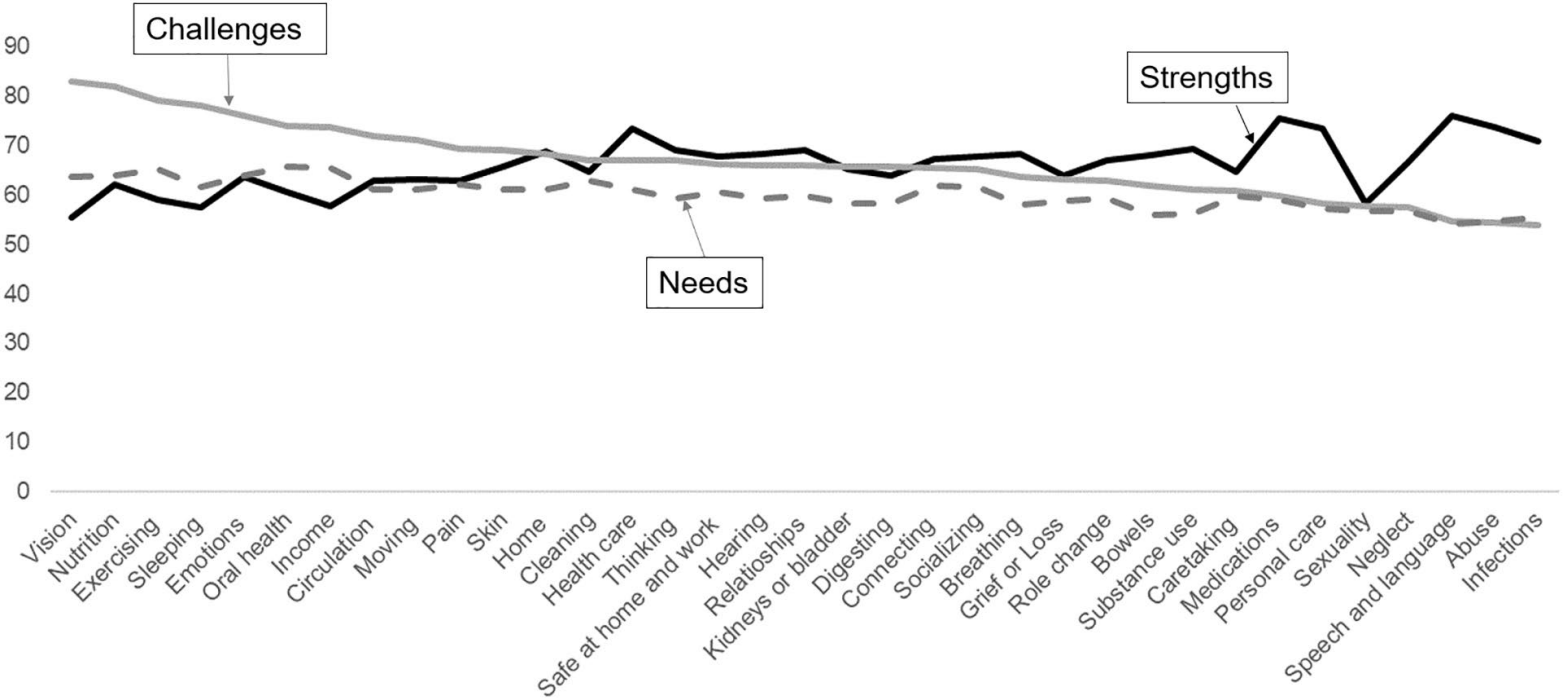
Percentage of challenges, strengths, and needs for adults 45 and older.

Following the pre‐processing stage, the exploratory data analysis revealed that the majority of participants (*N* = 988) were ages 45–64 (64.2%), female (57.5%), white (68.3%), Non‐Hispanic/Non‐Latino (79.3%), and married (55.3%). Overall, the most frequent Strength was *Speech and Language* (76.1%), Challenge was in *Vision* (83.1%), and Need was Hands‐on care in *Oral health* (65.8%). We use a parallel line graph to visualize percentages across all concepts sorted by most common Challenges (Figure 4).

Upon completion of machine learning analysis, data pre‐processing, several significant findings emerged across different groups. First, the *Thinking* group (F‐1 Score = 0.917) had 11 statistically significant challenges. The Challenge with the highest odds ratio (OR = 35.9) was *Breathing* (My Body), “need help to cough and spit out mucus.” The odds ratio indicates those with at least one *Thinking* challenge are 35 times more likely to have an association with the *Breathing* challenge “need help to cough and spit out mucus.” The *Moving* group (F‐1 Score = 0.905) had nine statistically significant challenges. The Challenge with the highest odds ratio (OR = 27.7) was *Pain* (My Body) “heart is racing and breathing is fast because of pain.” The *Emotions* group (F‐1 score = 0.921) had four statistically significant challenges. The Challenge with the highest odds ratio (OR = 15.7) was *Thinking* (My Mind and Networks) “hard to concentrate.” The Sleeping group (F‐1 score = 0.9) had four statistically significant challenges. The Challenges with the highest odds ratio (OR = 7) was *Medications* (My Self‐care) “need better system for taking meds” (Table 2).

**TABLE 2:** Statistically significant Challenges per Concept Group.

Domain	Concepts	Challenges	*p*‐value	OR
Thinking Concept Group (*n* = 633)				
My Body	Breathing	Need help to cough and spit out mucus	0.01	35.9
My Self‐care	Medications	Need better system for taking meds	0.002	27.6
My Body	Moving	Not coordinated	0.047	13.2
My Body	Circulation	Hard to find or weak pulses	0.025	10.7
My Living	Home	Unsafe or too steep stairs	0.026	4.8
My Body	Kidneys or bladder	Hard to get to the bathroom in time	0.035	4.5
My Mind and Networks	Emotions	Hard to manage my stress	0.007	4
My Body	Hearing	Hard to hear in crowds	0.0004	3.7
My Living	Cleaning	Bugs, rats, mice, squirrels, pests	0.024	3.6
My Body	Moving	Weak muscles	0.043	2.8
My Mind and Networks	Socializing	No hobbies or clubs	0.029	2.7
Moving Concept Group (*n* = 683)				
My Body	Pain	Heart is racing and breathing is fast because of pain	0.013	27.7
My Body	Pain	Hard to keep my face from showing I have pain	0.017	24.8
My Body	Circulation	Swelling	0	9.2
My Body	Pain	Hard to move because of pain	0.012	6
My Body	Pain	Restless because of pain	0.021	5.6
My Body	Breathing	Stuffed up nose or sinuses	0.004	3.6
My Self‐care	Exercising	do not like my exercise plan	0.004	2.6
My Body	Pain	Having pain	0.013	2.4
My Body	Vision	Hard to see things up close	0.039	0.4
Emotions Concept Group (*n* = 557)				
My Mind and Networks	Thinking	Hard to concentrate	0.002	15.7
My Mind and Networks	Grief or Loss	My grief or loss differs from others	0.022	7.9
My Mind and Networks	Grief or Loss	Hard to cope with my grief or loss	0.017	5.9
My Self‐care	Exercising	Hard to exercise the right amount	0.025	2.6
Sleeping Concept Group (*n* = 544)				
My Self‐care	Medications	Need better system for taking meds	0.01	7.4
My Body	Oral health	Crooked teeth or poor bite	0.042	3.9
My Body	Kidneys or bladder	Wake too often at night to go to the bathroom	0.004	2.5
My Mind and Networks	Emotions	Tired	0.039	1.7

### Examine differences strengths and needs (Aim 2)

The *Thinking* group had the highest average Strengths [66.5% (SD = 3.9)] followed by the *Moving* group [66.0% (SD = 4.6)], *Sleeping* group [60.6 (SD = 4.9)], and *Emotions* group [56.1% (SD = 3.8)]. All groups had statistically significant differences in Strengths (*p* < 0.001) except for Thinking/Moving group (*p* = 0.634). The *Thinking* group had the highest average Needs [83.6% (SD = 2.1)], followed by the *Moving* group [79.0 (SD = 2.7)], *Emotions* group [72.6% (SD = 3.1)], and *Sleeping* [70.4% (SD = 2.0)] and all groups had statistically significant differences in Needs (*p* < 0.01). All four groups reported Strengths in *Cleaning*, *Speech and language*, *Health care*, *Medications*, *Abuse* (meaning no abuse), and *Personal care*. Across all groups the most frequently reported Need was Hands‐on Care in *Cleaning*, *Connecting*, *Emotions*, *Exercising*, *Income*, *Oral Health*, and *Pain* (Table 3).

**TABLE 3:** Top 10 strengths and needs for each concept group.

Strengths			Needs			
Domain	Concept	%	Domain	Concept	Need	%
Thinking (*n* = 633)						
My Living	Cleaning	73.14	My Living	Income	Hands‐on Care	56.24
My Mind and Networks	Role change	72.83	My Living	Cleaning	Hands‐on Care	53.24
My Mind and Networks	Socializing	72.51	My Body	Oral health	Hands‐on Care	52.61
My Mind and Networks	Speech and language	72.51	My Mind and Networks	Socializing	Hands‐on Care	50.08
My Mind and Networks	Relationships	70.77	My Body	Pain	Hands‐on Care	49.76
My Self‐care	Health care	70.62	My Mind and Networks	Emotions	Hands‐on Care	49.61
My Self‐care	Medications	70.62	My Self‐care	Exercising	Hands‐on Care	49.29
My Mind and Networks	Abuse	70.3	My Mind and Networks	Connecting	Hands‐on Care	48.5
My Self‐care	Personal care	70.3	My Mind and Networks	Personal care	Hands‐on Care	48.5
My Living	Home	69.04	My Body	Thinking	Hands‐on Care	48.03
Emotions (*n* = 557)						
My Living	Cleaning	63.38	My Living	Income	Hands‐on Care	47.04
My Mind and Networks	Speech and language	63.2	My Living	Cleaning	Hands‐on Care	44.52
My Self‐care	Health care	61.04	My Body	Oral health	Hands‐on Care	44.34
My Mind and Networks	Abuse	60.68	My Body	Pain	Hands‐on Care	42.73
My Self‐care	Medications	60.5	My Mind and Networks	Emotions	Hands‐on Care	42.37
My Mind and Networks	Personal care	60.32	My Living	Home	Hands‐on Care	41.29
My Mind and Networks	Role change	59.61	My Mind and Networks	Connecting	Hands‐on Care	41.29
My Mind and Networks	Socializing	59.25	My Self‐care	Exercising	Hands‐on Care	41.29
My Mind and Networks	Relationships	59.25	My Self‐care	Personal care	Hands‐on Care	40.75
My Body	Hearing	58.89	My Self‐care	Health care	Hands‐on Care	40.39
Moving (*n* = 683)						
My Mind and Networks	Speech and language	74.23	My Living	Income	Hands‐on Care	53.15
My Mind and Networks	Socializing	72.47	My Living	Cleaning	Hands‐on Care	51.68
My Self‐care	Medications	72.47	My Body	Oral health	Hands‐on Care	50.22
My Mind and Networks	Abuse	71.74	My Body	Pain	Hands‐on Care	47.73
My Self‐care	Health care	71.16	My Self‐care	Exercising	Hands‐on Care	47.14
My Living	Cleaning	71.01	My Mind and Networks	Socializing	Hands‐on Care	46.56
My Mind and Networks	Role change	70.86	My Mind and Networks	Emotions	Hands‐on Care	46.41
My Mind and Networks	Relationships	70.86	My Self‐care	Personal care	Hands‐on Care	46.41
My Self‐care	Personal care	70.57	My Mind and Networks	Connecting	Hands‐on Care	46.12
My Body	Hearing	69.4	My Self‐care	Health care	Hands‐on Care	45.53
Sleeping (*n* = 544)						
My Mind and Networks	Speech and language	70.59	My Body	Oral health	Hands‐on Care	47.24
My Mind and Networks	Abuse	68.38	My Living	Income	Hands‐on Care	47.06
My Self‐care	Medications	67.28	My Living	Cleaning	Hands‐on Care	46.88
My Self‐care	Personal care	66.73	My Body	Pain	Hands‐on Care	43.75
My Living	Cleaning	65.63	My Living	Home	Hands‐on Care	43.57
My Self‐care	Health care	65.07	My Mind and Networks	Socializing	Hands‐on Care	43.38
My Self‐care	Substance use	64.52	My Mind and Networks	Emotions	Hands‐on Care	43.38
My Living	Home	64.15	My Self‐care	Health care	Hands‐on Care	43.01
My Body	Infections	63.97	My Self‐care	Exercising	Hands‐on Care	42.65
My Mind and Networks	Connecting	63.79	My Mind and Networks	Connecting	Hands‐on Care	41.91

## DISCUSSION

This retrospective cross‐sectional analysis applied machine learning methods to analyze de‐identified whole person health data from the MSMH app, exploring healthy aging in adults aged 45 and older. Despite facing numerous challenges and needs, adults in this age group display many Strengths. This finding is consistent with priorMSMH research showing similar trends in other populations (Austin, Mathiason, & Monsen, 2022).

The four groups varied in the number of statistically significant Challenges and in average Strengths and Needs across all domains. This variation is consistent with current literature, which highlights older adults have wide‐ranging co‐morbidities, underscoring the need for whole person approaches to identify and manage these diverse health challenges (Langevin et al., 2024). A novel finding was that when an individual had at least one *Thinking* challenge, they had a broader range of health Challenges compared to the other groups. This finding emphasizes adults with cognitive health challenges are more likely to have multiple co‐occurring physical issues (e.g., respiratory) but also social challenges (e.g., income and mental health) and aligns with previous research demonstrating the complex array of co‐existing health challenges (Gaugler et al., 2023). Further research is needed to confirm these findings in other adult populations.

While Strengths and Needs varied across all concept groups and domains, there were similarities across all four groups. All groups reported Strengths in *Cleaning*, *Healthcare*, and reports of no *Abuse* were encouraging. Furthermore, the *Thinking* group had the highest average Strengths and Needs compared to the other groups. The observation that adults with mild cognitive challenges exhibit greater Strengths and Needs compared to other groups aligns with current literature, which indicates these adults possess various strengths and have a wide range of health needs (Fuller & Huseth‐Zosel, 2020; Meléndez et al., 2018). Healthy aging research recommends a whole person approach that includes identifying social drivers of health and to build on an individuals' strengths (Langevin et al., 2024). The use of whole person health data, from MSMH, has potential to provide actionable data that can support a more personalized approach to healthy aging. For example, by explicitly incorporating an individual's strengths as part of a care plan, these strengths can be intentionally used to mitigate health challenges and needs. Our future research will explore ways to incorporate both self‐reported strengths and needs into clinical care workflow for adults, particularly for those with cognitive challenges.

The ability to apply machine learning methods to structured consumer‐generated whole person health data facilitates new approaches to data analysis and generates personalized approaches to healthy aging (Bakken, 2021a). This systematic exploratory data analysis process enabled the examination of outputs at each step to ensure results were meaningful and clinically relevant. The structured data from MSMH, based on a standardized terminology, are multidimensional and may have inherent relationships within the data. Recognizing potential inherent relationships in the data can predetermine specific machine learning methods best applied to structured data (e.g., PCA, Random Forest). Nurses and nurse informaticians are poised to lead data‐driven artificial intelligence research that has the potential to generate innovative consumer‐centric solutions to transform healthcare (von Gerich et al., 2022). Nurse focused groups leading these efforts and focusing on sharing artificial intelligence and methodology such as machine learning knowledge currently exist (Ronquillo et al., 2021).

There are limitations with this study. This study used retrospective cross‐sectional data of adults aged 45 years and older. As is common with cross‐sectional studies, there may be inherent biases in the data and thus not generalizable to all adults in the same age group. Future research will explore MSMH longitudinal data collection to expand the capacity to track whole person health, including strengths, over time. The MSMH data are based on a standardized structured language, the Omaha System, and have inherent relationships within the data. The inherent relationships required us to make strategic data analysis decisions to overcome the multi‐dimensionality of the data (Pailaha, 2023).

This research notably impacts nurses' involvement in research, practice, and policy, specifically as it relates to AI. Nurses play a critical role in AI research, particularly through the adoption of machine learning methods. This can empower nurses to lead innovative science, enhance knowledge generation, and advance patient care forward (Ronquillo et al., 2021; Topaz & Pruinelli, 2017). This research demonstrates how nurses and nurse informaticians can actively participate in all phases of the AI driven research, including development, implementation, and evaluation. For example, in this study nurses provided clinical expertise to the analysis which were essential to ensure the findings were accurate and clinically relevant. The role of nurses at each phase, initial design, deployment, and ongoing assessment, is necessary to ensure that these technologies are practical, ethically minded, user‐friendly, and tailored to meet the real‐world needs of patient care. In clinical practice, nurses are needed to partner with AI developers and share their firsthand experience in developing and adopting tools that can benefit nursing practice and enhance clinical outcomes. From a policy perspective, it is imperative that nurses are involved in AI policy development to address ethical considerations, data security, and workforce readiness. This aligns with national initiatives promoting the ethical use of AI, where nurses and nurse informaticians already provide critical input, (Algorithmic Justice League, 2024; American Nurses Association (ANA), 2022). Nurses play a pivotal role at all stages of AI technology integration, ensuring its effectiveness. Therefore, it is vital for nurses to actively contribute their expertise to advance patient care, optimize workflows, and promote healthcare excellence (Pailaha, 2023).

## CONCLUSION

This research provides a framework for applying machine learning methods to consumer‐generated whole person health and resilience data. By employing these methods, we have identified unique associations and novel insights that are applicable to adults with specific health challenges, such as cognitive challenges, and to promote healthy aging. The next phase of this research involves developing and testing personalized interventions derived from machine learning‐enhanced data to support data‐driven whole person health strategies. This research highlights nurses are well positioned to lead AI initiatives and leverage tools, such as machine learning, to transform data into practical clinical interventions.

## CONFLICT OF INTEREST STATEMENT

The authors declare no conflict of interest.

## CLINICAL RESOURCES

Nursing and Artificial Intelligence Leadership (NAIL) Collaborative: https://www.nailcollab.org/. ANA Position Statement on the Ethical use of Artificial Intelligence in Nursing Practice: https://www.nursingworld.org/~48f653/globalassets/practiceandpolicy/nursing‐excellence/ana‐position‐statements/the‐ethical‐use‐of‐artificial‐intelligence‐in‐nursing‐practice_bod‐approved‐12_20_22.pdf. The White House Executive Order on the Safe, Secure, and Trustworthy Development and Use of Artificial Intelligence: https://www.whitehouse.gov/briefing‐room/presidential‐actions/2023/10/30/executive‐order‐on‐the‐safe‐secure‐and‐trustworthy‐development‐and‐use‐of‐artificial‐intelligence/.

## Data Availability

The data that support the findings of this study are available from the corresponding author upon reasonable request.
